# Extraction of the Proximal Phalanx: A New Option in Surgical Treatment of the Crossover Second Toe

**DOI:** 10.1155/2020/3901458

**Published:** 2020-01-20

**Authors:** Željko Jeleč, Tomislav Gjurašin, Ana Vuković Pirkl, Gordan Rujevčan

**Affiliations:** ^1^St. Catherine Specialty Hospital, Zabok 49210, Croatia; ^2^University North, Varaždin 42000, Croatia; ^3^School of Medicine, University of Osijek, Osijek 31000, Croatia; ^4^General Hospital “Dr. Ivo Pedišić”, Sisak 44000, Croatia

## Abstract

One of the biggest and commonest problems that is seen and treated by foot and ankle surgeons is the deformity where the second toe crosses over the hallux. According to available literature, this is the first published case of extraction of the proximal phalanx due to crossover toe deformity. We present the case of a 64-year-old Caucasian female with a crossover second toe deformity of her left foot. Because of this deformity, she was completely disabled to wear normal shoes and she felt intensive pain in her front part of the foot. She underwent a total extraction of the proximal phalanx of the second toe. After the operation, she was very satisfied with the status of the operated foot and the final result of the surgical treatment. The procedure that we performed could be a good possibility for the treatment of crossover second toe deformity because we got a good functional and cosmetic result, the morbidity associated with more advanced reconstruction is avoided, and the rehabilitation period was short. Patient satisfaction was high, and complications were minimal.

## 1. Introduction

One of the biggest and commonest problems that is seen and treated by foot and ankle surgeons is the deformity where the second toe crosses over the hallux. In a patient's clinical presentation, a crossover toe can be sole deformity of the foot or could be a part of complex foot deformity with the hallux valgus, hallux rigidus, or hammertoe [1, 2]. The first mention of the term “crossover toe” was in 1986 during Coughlin's lecture at the 16th annual meeting of the American Orthopaedic Foot and Ankle Society (AOFAS) [3]. According to Kaz and Coughlin [2], the maximal incidence of crossover second toe deformity is in women who are older than 50 years. A crossover second toe can have a posttraumatic or idiopathic aetiology. Posttraumatic aetiology is rarer and in most cases is a result of acute trauma of the first metatarsophalangeal (MTP) joint which includes forced hyperextension [2, 4, 5].

The idiopathic form is more common, and the main symptom is inflammation whose cause is unknown. Kaz and Coughlin reported that the cause of increased load on the second MTP joint could be a long second metatarsal bone or hypermobility of the first ray. Other possible reasons could be genetic predisposition or static disorders of the foot [2]. Associated with a crossover second toe, it is possible to find a destruction of other anatomic structures like a plantar plate [6].

There are a few studies which showed that deformities of the toes have a great impact on other parts of the musculoskeletal system and balance, especially in elderly people [7, 8]. Mentioned studies showed that shape of the foot is also very important for postural stability. According to these studies, the left leg shows a greater impact on stability than the right one.

In their paper published in 1999, Haddad et al. gave the first classification of a crossover second toe [9]. They divided this deformity into 4 groups, according to the degree of the dislocation of the second MTP joint.

The treatment of this deformity can be conservative or operative. In stages 1-3 (when the second MTP joint is not completely dislocated), it is indicated to start with conservative treatment (metatarsal pad, nonsteroidal anti-inflammatory drugs, and orthotics). If the second MTP joint is completely dislocated (stage 4 according to Haddad) and in case of unsuccessful conservative treatment, operative management is indicated. A number of different techniques have been described for this approach, many of which are successful in selected circumstances, but finding a universal approach has been somewhat impossible.

In general, operative treatment could be divided into soft tissue procedures, bone procedures, and their combinations. Isolated soft tissue procedures are reserved for mild deformities; in most cases, dorsal and medial capsular release is performed, somewhere with lateral capsular tightening. In case of severe deformity, a good option is shortening osteotomy of the second metatarsal bone, according to Coughlin and Mann [10]. The Weil osteotomy is a very successful solution.

Amputation of the second toe can also be one of the options. According to Gallentine and DeOrio [11], this procedure is acceptable in elderly patients for complaints of pain related solely to the hammertoe.

In the article “Second metatarsophalangeal joint instability (crossover toe),” it gave a very informative and detailed review of aetiology and classification, as well as treatment options of the crossover second toe deformity [12].

The purpose of this case report is to present for the first time a novel method in the treatment of the complex deformity of the forefoot. The objective is to introduce our result to readers and to compare our method with others. According to available literature, this is the first patient treated in that way because of crossover second toe deformity.

## 2. Case Presentation

We present the case of a 64-year-old Caucasian female who was treated at our department because of the deformity of her left foot. Because of this deformity, she was completely disabled wearing ready-made shoes. She felt pain in the forefoot on dayly basis. Medial side of the thumb and dorsal side of the second toe were sometimes inflammed including secretion. Four years earlier, an orthopaedic surgeon advised her surgery, which among other things included the amputation of the second toe, and the patient had not consented. On several occasions, she underwent physical therapy consisting of analgesic and anti-inflammatory procedures and exercises to strengthen the muscles of the foot and lower leg. The physical therapy she performed had no positive effect. In her medical history, there were no serious diseases; she was being treated for hypertension at the time.

**Figure 1:**
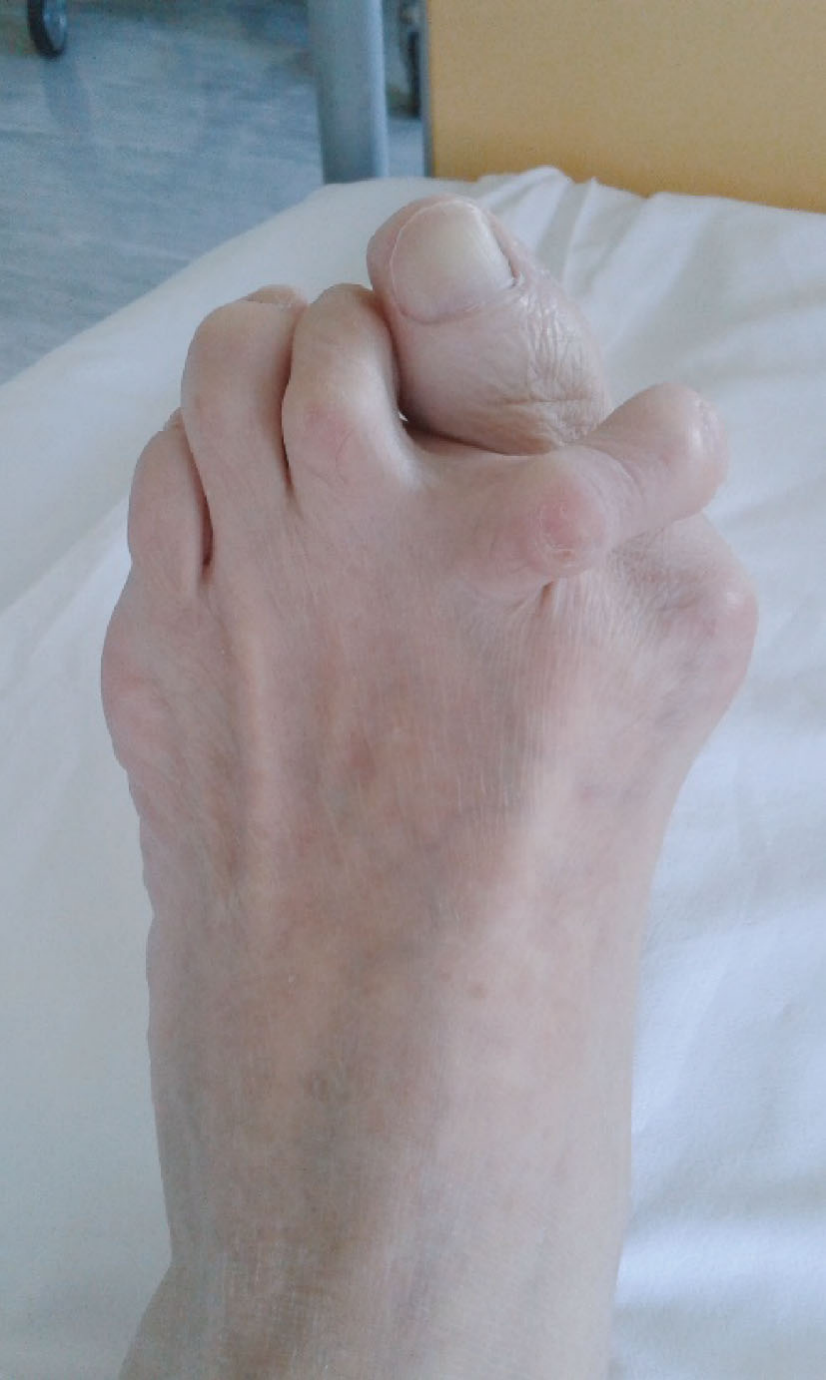
Photograph of the patient's left foot before the operation.

Clinical examination verifies the pronounced deformation of the left forefoot in terms of the valgus position of the thumb, crossover second toe, and third claw toe, with a distinct contracture of involved joints (Figure 1).

**Figure 2:**
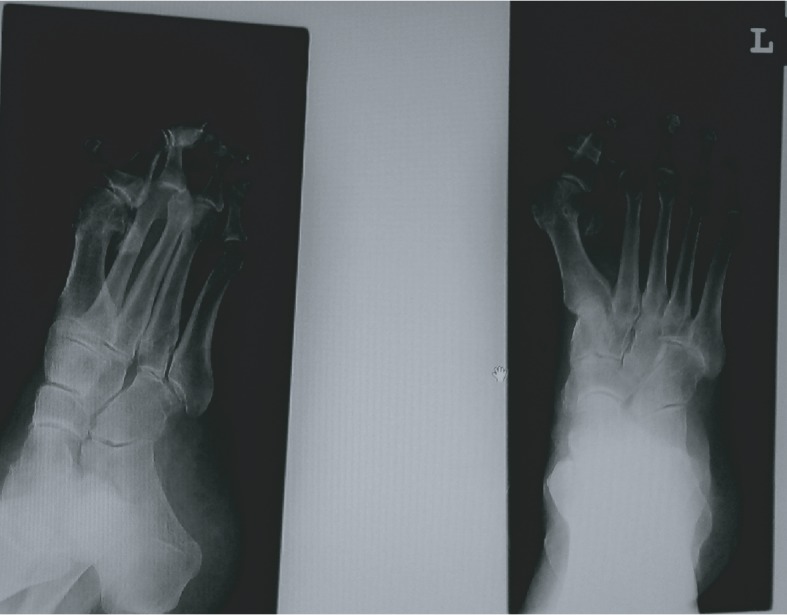
X-ray of the patient's left foot before the operation.

She had painful plantar callosity in the projection of the third metatarsal head. The X-ray of her left foot showed a severe deformity of the second toe and its overlapping and complete dislocation of the second and third metatarsophalangeal joints. X-ray also showed a high degree of valgus position of the thumb with severe arthrosis of the first MTP joint with medial osteophytes (Figure 2).

**Figure 3:**
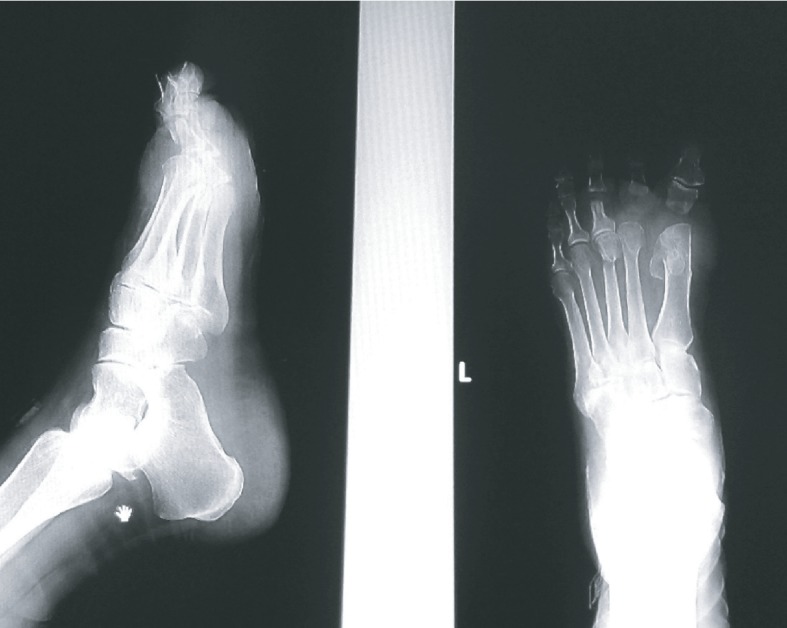
X-ray of the patient's left foot at the first postoperative day.

**Figure 4:**
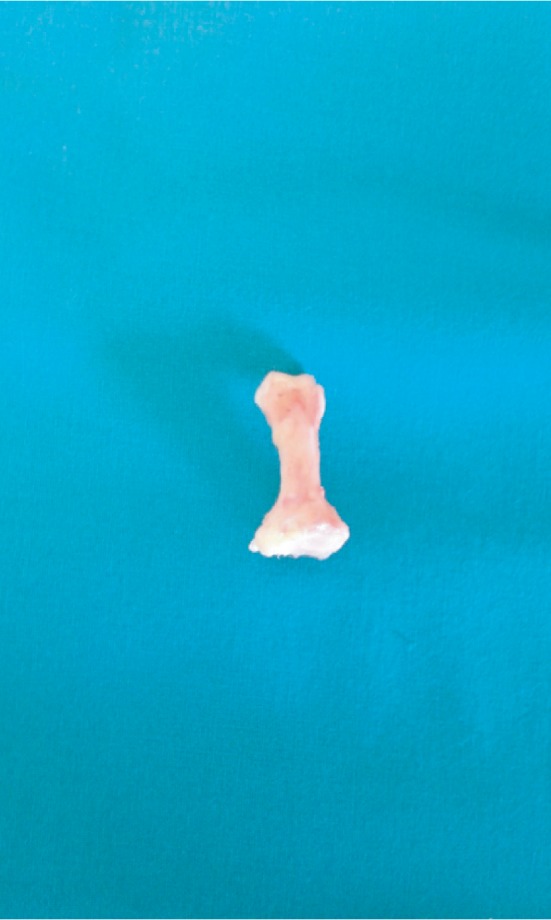
Photograph of the extirpated proximal phalanx of the second toe.

After preoperative preparation, we performed the surgery in regional anesthesia (spinal block) with antibiotic prophylaxis. On the thumb, planned resection of the base of the proximal phalanx is made (classic intervention by Keller). Due to the extreme rigid deformities of the second toe and because of complete dislocations in the second MTP joint, we made a decision to do complete extraction of the proximal phalanx of the second toe. On the third toe, we did a resection of the head of the proximal phalanx, and actually we performed resection arthroplasty of the third MTP joint. Because of the painful plantar callosity in the projection of the third metatarsal head, we performed an oblique subcapital osteotomy of the third metatarsal bone (procedure by Helal) (Figures 3 and 4).

We had an idea to do the same operative procedure on the second metatarsal bone, but because of the risk of circulatory collapse, we made a decision not to do that procedure in the same time.

**Figure 5:**
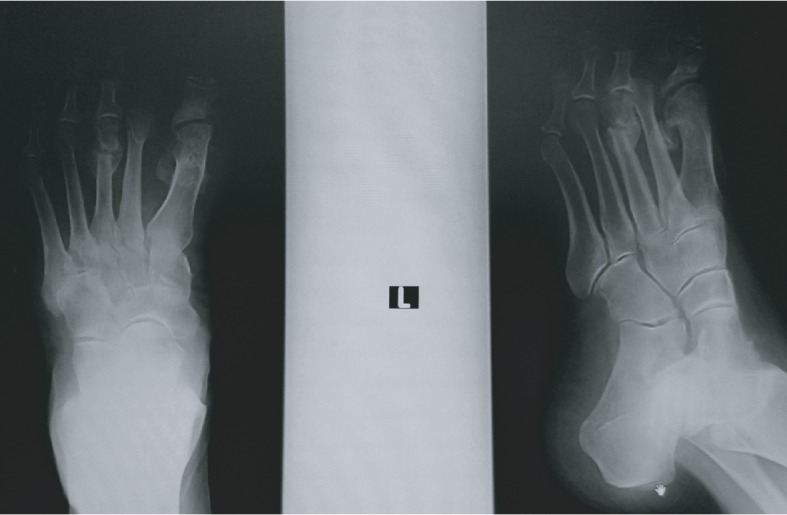
X-ray of the patient's left foot three months after the operation.

**Figure 6:**
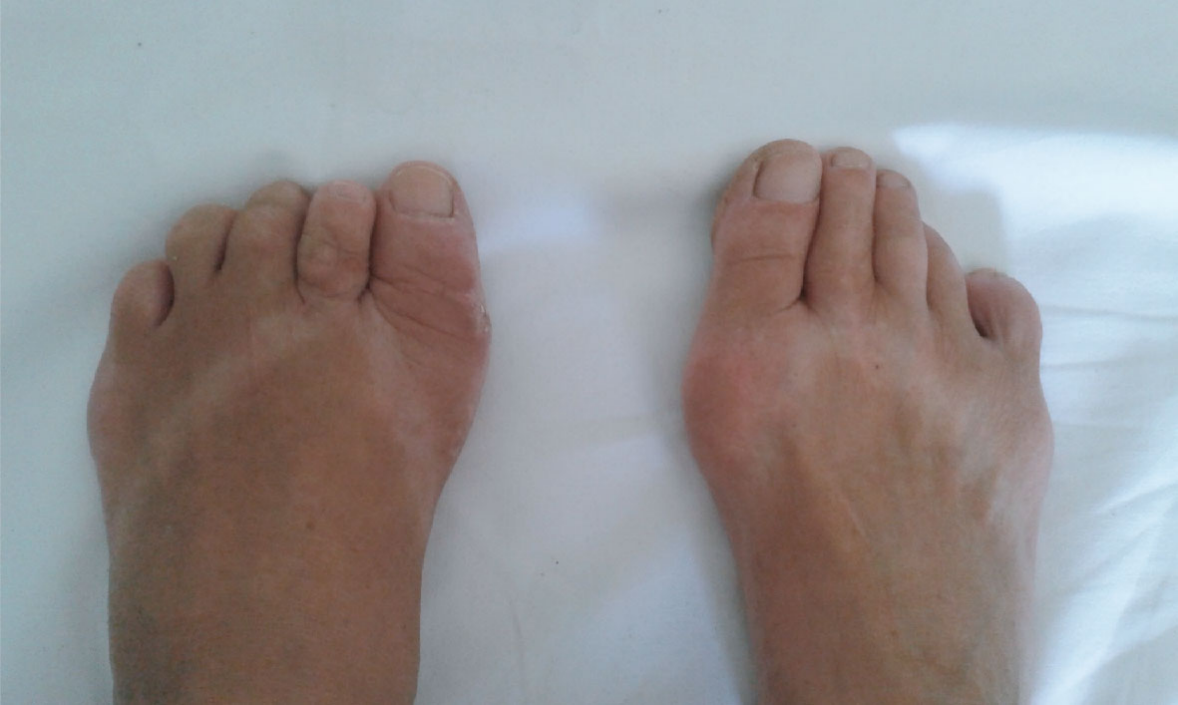
Photograph of both of the patient's feet three months after the operation.

The postoperative recovery was normal. From the first postoperative day, full weightbearing with a maximum load on the left heel was allowed. On the second postoperative day, she was discharged to home care, and on the twelfth postoperative day, the sutures were removed. Two weeks after surgery, full weightbearing without any restrictions (to the pain tolerance) was allowed. Physical therapy started two weeks after surgery, lasted three weeks, and was performed three times a week. The therapy consisted of stretching the toes and ankles to achieve a normal range of motion, anti-inflammatory procedures and lymphatic drainage in order to reduce swelling of the forefoot, and muscle strengthening exercises. A control radiograph showed the correct position of the thumb and the second and third toes, including the place of the third metatarsal osteotomy (Figure 5); three months after surgery, she was able to wear all kinds of shoes including thong slippers (Figure 6).

The only complaint about her left foot was a mild plantar pain in the projection of the second metatarsal head after walking on longer distances. Despite these subjective symptoms, she was very satisfied with the status of the operated foot and the final result of operative treatment.

## 3. Discussion

The treatment of the second crossover toe is a challenge for the patient and the orthopedic surgeon. Many different operating procedures have been described in the literature, of which some have been mentioned in Introduction including their basic principles.

In our case, we had a patient with huge deformity of her left foot and she had complete dislocation of the second MTP joint; according to Haddad et al. [9], it was stage 4 deformity. Due to this deformity, she was completely disabled to wear normal shoes and she felt intensive pain in the front part of her foot. Because of severe deformity (stage 4) and failed physical therapy, surgical treatment was indicated.

As we mentioned before, very often associated with the crossover toe deformity, it is possible to find a rupture of the plantar plate [6]. The plantar plate has an important role in metatarsophalangeal stability, and many authors described their techniques for reconstruction of the plantar plate [13–15]. Reconstruction of the plantar plate is a very helpful and successful procedure in mild-to-moderate stages of deformity, where we have instability of the MTP joint. In severe stages with complete MTP joint dislocation, reconstruction of the plantar plate without some bone procedure will not be sufficient.

A very popular technique in surgical treatment of lesser metatarsals is the standard Weil osteotomy, first time performed in 1985 [16]. Originally, this technique was reserved for patients with extremely long second metatarsal bone. Later, indications were widened especially on patients with some lesser toe deformities [17]. In our case, due to a huge degree of the luxation of the second MTP joint, we made a decision not to do this type of surgery. It was clear that it is not possible to solve the patient's problem only with the Weil osteotomy. An additional procedure like a plantar plate reconstruction would prolong the time for recovery and increase the possibility for some complication, which we wanted to avoid. We have to emphasize that our patient had a problem with the great and third toe also. A simultaneous surgical procedure on three rays is very hard for a patient, especially an elderly person. That was the reason more for our decision to perform a little bit simpler procedure.

As we mentioned before, amputation of the second toe could also be an option for the treatment of this kind of deformity. According to Sundaram and Walsh, amputation is acceptable in patients older than 70 if the first ray is not involved [18]. Gallentine and DeOrio [11] and Myerson [19] made very similar conclusion independently.

Resection arthroplasty of the lesser MTP joint is a good indication for hammertoe deformity [20]. According to the same authors, resection arthroplasty could be performed for crossover toe deformity also. In our case, we did resection arthroplasty for deformity of the third toe. Because of the degree of the deformity of the second toe, our estimation was that resection arthroplasty will not be successful.

Our patient wanted a painless foot with an acceptable cosmetic effect. Because of the severe deformity, there was a real danger of complications associated with some advanced reconstruction of her forefoot. The direct consequence of some advanced reconstruction is a prolonged time of the rehabilitation period, which we wanted to avoid.

One of the interventions that we did was a complete extraction of the proximal phalanx of the second toe. According to available literature, this is the first patient treated with the extraction of the proximal phalanx because of a crossover second toe. From that reason, we could not compare our result with the results of others who used the same procedure.

With this procedure, we achieved two main goals: our patient got a painless and esthetic acceptable foot and we avoided potential complications and prolonged rehabilitation. We strongly believe that this procedure has its place in operative treatment of crossover second toe deformity. For an optimal result of the surgery, it is important to do good preoperative planning and to use a very precise and thorough operative technique. The last but not the least, patient compliance is very important. The patient should be informed about the procedure, and the patient's expectations should be reasonable.

## 4. Conclusion

We believe that the procedure that we performed could be a good option for the treatment of crossover second toe deformity. In our opinion, the main advantage of our approach for the treatment of this deformity is the fact that we got a good functional and cosmetic result, the morbidity associated with more advanced reconstruction is avoided, and the rehabilitation period was short. Patient satisfaction was high, and complications were minimal.
